# The combined effects of growth and maturity status on injury risk in an elite football academy

**DOI:** 10.5114/biolsport.2024.129472

**Published:** 2023-08-08

**Authors:** Xabier Monasterio, Sean P. Cumming, Jon Larruskain, David M. Johnson, Susana M. Gil, Iraia Bidaurrazaga-Letona, Jose A. Lekue, Gontzal Diaz-Beitia, Juan M. Santisteban, Sean Williams

**Affiliations:** 1 Department of Physiology, Faculty of Medicine and Nursing, University of the Basque Country (UPV/EHU), Leioa, Spain; 2 Medical Services, Athletic Club, Lezama, Spain; 3 Department for Health, University of Bath, Bath, United Kingdom

**Keywords:** Youth, Adolescence, Football, Epidemiology, Height, Injury prevention

## Abstract

This study aimed to explore the interaction between growth rate on specific injury incidence and burden on pre-, circa- and post-peak height velocity (PHV) periods. Injury and stature data collected during the 2000–2020 seasons in an elite football academy were retrospectively analysed. Only players with height measurements from childhood until the attainment of adult height were included in the study (N = 84). Growth data were smoothed using a cubic spline to calculate daily growth rate and height. Growth rate was categorised into three groups: fast (> 7.2 cm/year), moderate (3.5–7.2 cm/year) and slow (< 3.5 cm/year). Percentage of observed adult height was used to classify players as pre-PHV (< 88%), circa-PHV (88–95%) or post-PHV (> 95%). Overall and specific injury incidence and burden and rate ratios for comparisons between growth rate groups were calculated on pre-, circa- and post-PHV periods, separately. Overall injury incidence and burden were greater in pre-PHV players with quicker growth rates compared to players growing moderately and slowly. All in all, players with more rapid growth-rates were at higher risk for growth-related injuries in all pre-, circa- and post-PHV periods. Post-PHV, the incidence and burden of joint/ligament injuries were 2.4 and 2.6-times greater in players growing slowly compared to players growing moderately. Practitioners should monitor growth rate and maturity status and consider their interaction to facilitate the design of targeted injury risk reduction strategies.

## INTRODUCTION

Injuries can result in long absences from training and matches in academy football players, reducing the opportunity for players to develop their fitness and skills [1]. Consequently, injuries negatively impact players’ academy progression [2]. Injuries occurring during childhood and adolescence can also result in long-term consequences, making players more susceptible to future injuries and long-term health risks (e.g., osteoarthritis) [3]. Thus, injury risk reduction strategies in youth footballers are vital to ensure the development of healthy youth players and ensure the long-term health of the professional football players.

During adolescence, players experience a marked and rapid period of somatic growth [4], leading to evident changes in limb length, limb mass, and moments of inertia [4]. As a consequence of these changes, temporary delays or regressions in sensorimotor mechanisms and motor control may be observed during this period [5], adversely impacting injury risk. Accordingly, the International Olympic Committee [6] and league governmental bodies (e.g., English Premier League) [7] have highlighted the importance of assessing and monitoring inter-individual variations in growth and maturity.

Growth rate is used to describe changes of a physical dimension (e.g., standing height) over a given time [4]. During the adolescence there is an increase in the rate of growth, with highest point known as peak height velocity (PHV). PHV is observed around the age of 13–14 years in boys, reaching maximal growth rates of 5.6–12.4 cm/year [4]. To date, a limited number of studies in youth football academies have investigated the influence of adolescent growth rates upon injury [8–11]. Kemper et al. [8] and Rommers et al. [9] observed that injured male adolescent players had a higher rate of growth compared to non-injured players. Similarly, Johnson et al. [11] reported that players with a rate of growth rate > 7.2 cm/year were more likely to be injured than players growing less than 7.2 cm/year. Not only that, but they also showed that there was a linear increase in injury risk associated with growth rate [11]. Concerning the risk for specific types of injuries, Wik et al. [12] found that overall growth rate was associated with a greater risk of bone and growth plate injures in adolescent athletics.

Biological maturation is a separate and more complex concept. The level of biological maturation at a given point, defined as maturity status, indicates where along the process towards a mature state a given tissue or organ system (somatic, skeletal, or sexual) is at the time of measurement [4]. The percentage of adult height at the time of observation is an indicator of somatic maturity that is increasingly used in youth athletes and allows to easily classify players as pre- (< 88%), circa- (88–95%), or post-PHV (> 95%) [13]. Available research has suggested that injury incidence and burden is higher in circa-PHV compared to pre-PHV period [14], whilst a recent study has found that the occurrence of specific injuries varies according to the percentage of adult height [15]. Growth-related injuries were more frequent in percentages around PHV (91.2%) while muscle and joint/ligament injuries were more common in post-PHV [15]. Interestingly, growth-related injuries occurred from distal to proximal body regions, following the pattern of growth and maturation [15]. As a result, growth-related injuries occurring on distal segments (e.g., Sever´s and Osgood-Schlatter´s disease) peaked in pre- and circa-PHV periods while proximal injuries (e.g., spondylolysis) peaked in post-PHV [15].

To date, only one study has analysed the interaction between growth-rate and maturity status upon injury risk. Johnson et al. [11] showed that there is an increase in estimated injury likelihood at a high growth rate circa-PHV. However, they found an increase in estimated injury burden likelihood at a lower growth rate and a higher percentage of predicted adult stature (post-PHV). Despite the novel results found by Johnson et al. [11], this study has potential limiting factors. First, the data were recorded over a single season period, making it impossible to follow individuals during a sufficient interval of time to model individual growth curves and account for the non-linear characteristic of growth [16]. Further, the Khamis-Roche equation was used to estimate adult height. If measured accurately, this equation is reported to predict adult height to within 2.2 and 5.3 cm for the 50^th^ and 90^th^ percentile, respectively; therefore, the use of Khamis-Roche equation might have led some players to be misclassified as pre-, circa- or post-PHV due to errors associated with the prediction [13]. Most importantly, this research did not study the interaction between growth-rate and injury risk of specific injuries in pre-, circa- and post-PHV periods. Considering that growth-rates [4] and injury patterns [15, 17] differ according to maturity status, studying the impact of growth-rate on specific injury risk in each period seems vital.

The present study builds upon the abovementioned limitations by using height and injury data recorded in an elite football academy over two decades. This permits a more accurate estimation of growth rate and percentage of the observed adult height of players and affords the opportunity to explore potential interactions between growth rate (cm/year) and risk for specific types of injuries (incidence and burden) in pre-, circa- and post-PHV periods, separately.

## MATERIALS AND METHODS

### Study design and participants

This retrospective analysis studied height and injury data recorded longitudinally for 20 consecutive seasons (2000–2020) in Athletic Club’s elite soccer academy whose professional male team plays in Spanish LaLiga. The academy has a team in each of the age-based levels or categories. In men, this includes U11, U12, U13, U14, U15, U16, U17, and U19 teams, in addition to 3^rd^ and 2^nd^ teams comprising 17–23-year-old players competing in the Spanish Fourth and Third Divisions, respectively. Among the 1123 players who were followed, only players who were ≤ U12 when they entered the academy and continued until they attained adult height were included in the study (n = 84) attempting to equally represent pre-, circa- and post-PHV periods.

The study was conducted in accordance with the National Health Council resolution (466/2012) and was approved by the Ethics Committee of the University of The Basque Country (UPV/EHU) (CEISH/340/2015). Written informed consent to use regularly collected data for research purposes was obtained from the players.

### Height measurement, growth-rate estimation, and maturity status assessment

Standing stature was measured by trained doctors at least twice annually using a portable stadiometer (Añó Savol, Spain). Participants stood barefoot with feet together and their head in the Frankfort plane. They were required to take a deep breath and hold their head still while measuring. Two of the four doctors worked in the academy during the entire study period, thereby reducing chance of bias. The intra-rater typical error of measurement for standing stature of these two doctors was 0.23 cm while the inter-rater error was 0.29 cm.

Growth rate was calculated as the change in stature over the change in time (cm/year). Growth data were smoothed using a Cubic spline. The spline would fit a curve across the whole time period using the multiple measurement points and subsequently, a growth rate and height per day could be estimated from this curve [18]. The calculation of the spline allowed an estimate of growth rate for each training/match day, which allowed the growth and maturation data to match with daily observations of daily training/match exposure.

Growth rate was categorised into three groups: fast (> 7.2 cm/year), moderate (7.2–3.5 cm/year) and slow (< 3.5 cm/year), based on previous literature [8, 11] and to achieve an approximately equal number of observations per group.

The percentage of observed adult height was used as a maturity status indicator [4]. A player was considered to have attained final height once growth-velocity was < 1 cm/year for one year [4]. The observed adult height allowed to calculate percentage of adult height using estimated height. Players were classified as: pre-PHV (< 88%), circa-PHV (88–95%) or post-PHV (> 95%) [11, 13].

### Injury definitions, exposure, and recording procedures

Time-loss injuries were recorded in the club’s online database by academy’s doctors when a player was unable to take part in full football training or match due to a physical complaint [19]. Absence days were calculated as the number of days elapsed between the initial injury date and the player’s return to full availability for training and matches [19].

From the 2007–2008 season onward, injuries were described following the International Federation of Association Football (FIFA) Consensus [19]. For each injury, the date of injury, injury type, session type, contact type and specific mechanism were reported. In the previous seasons, specific injury diagnosis and absence days of time-loss injuries were recorded. This allowed to categorise type of injuries (e.g., muscle injury) recorded before the publication of the FIFA Consensus. As the Consensus by Fuller et al. [19] did not explicitly consider growth-related injuries, the injury surveillance system was customised by adding a category for “growth-related injuries”, which were defined as “unique injuries not seen in adults but common in skeletally immature athletes (e.g., growth plate fractures, apophysitis, apophyseal avulsion fractures, and greenstick fractures)” [20]. Growth-related injuries were classified according to physical examination (e.g., pain at insertional points on palpation, passive movements and stretches, and active movements including resistance testing) and imaging diagnosis (ultrasound and/or magnetic resonance imaging). Two of the four doctors worked in the academy since the start of the study, thereby reducing the chance of bias, differences in injury interpretation, and changes in observation methods between doctors.

Daily exposure in matches and training sessions in available non-injured players was estimated based on the number and duration of matches and trainings, squad size and the number of players on the pitch in each category [21]. Players had 3 (U11–U12) or 4 (U13–Reserves) 90-minutes training sessions per week and played a match every weekend. Match length was 70 minutes for U11–U14, 80 minutes for U15–U16 and 90 minutes for older age-groups. The number of players on the pitch was 11 for all categories except for U11–U12, in which 7 players played in each team.

### Data analysis

Injury incidence (number of time-loss injuries/1000 hours) and injury burden (number of days lost/1000 hours) were calculated with 95% CI assuming a Poisson distribution [22]. Generalized linear mixed-effects models (GLMM) were used to compare incidence and burden between growth-rate groups (fast *vs*. moderate *vs*. slow) in each maturity status period (pre-, circa- or post-PHV) using a Poisson distribution and log-link function. The predictor variables were modelled as categorical fixed effects and player ID was included as a random effect to account for repeated observations. Statistical significance was accepted at p < 0.05 for incidences, while significant differences for injury burden were considered when the 95% confidence intervals did not overlap [23]. Bonferroni adjustments were performed to control the Type I error rate when making multiple comparisons. All analyses were performed using R version 4.1.2 (R Core Team 2021, R Foundation for Statistical Computing, Vienna, Austria).

## RESULTS

Player demographics, growth, and maturity data according to maturity status are presented in Table 1. There were 782 injuries and 162,314 hours of total exposure. The mean (SD) exposure for each player was 1932.3 (± 439.9) hours. The mean (SD) values for the percentage of observed adult stature and growth rate were 92.38 (± 6.64) % and 5.57 (± 3.35) cm/year, respectively. The overall injury incidence rate was 4.82 injuries per 1,000 hours (95% CI 4.49–5.17), the mean time-loss of injuries was 23 days (95% CI 21–26) and injury burden was 113 days absent per 1,000 hours (95% CI 105–121). Injury incidence, time-loss, and burden in each maturity status period are shown in Table 1.

**TABLE 1:** Stature, growth velocity, % of observed adult height, injury counts, exposure, incidence rates, mean severity, and injury burden according to maturity status.

Maturity status	Stature (cm)^a^	Growth velocity (cm/year)^a^	% of observed adult height^a^	Injury count (n)	Exposure (hours)	Injury incidence (per 1000 hours)^b^	Mean time loss (days)^b^	Injury burden (per 1000 hours)^b^
Pre-PHV	149.7 ± 6.0	5.8 ± 2.5	83.5 ± 2.6	147	51544	2.85 (2.43–3.35)	15.4 (12.6–18.2)	43.9 (37.3–51.6)

Circa-PHV	165.9 ± 6.3	7.7 ± 2.7	92.4 ± 2.4	234	40417	5.79 (5.09–6.58)	23.5 (19.7–27.3)	136.0 (119.6–154.6)

Post-PHV	176.8 ± 5.2	2.0 ± 1.9	98.4 ± 1.1	401	70353	5.70 (5.17–6.29)	26.5 (21.8–31–2)	151.1 (137.0–166.6)

a Anthropometrical variables are shown as mean ± SD.

b Incidence, severity, and injury burden are expressed with 95% confidence intervals

**FIG. 1:**
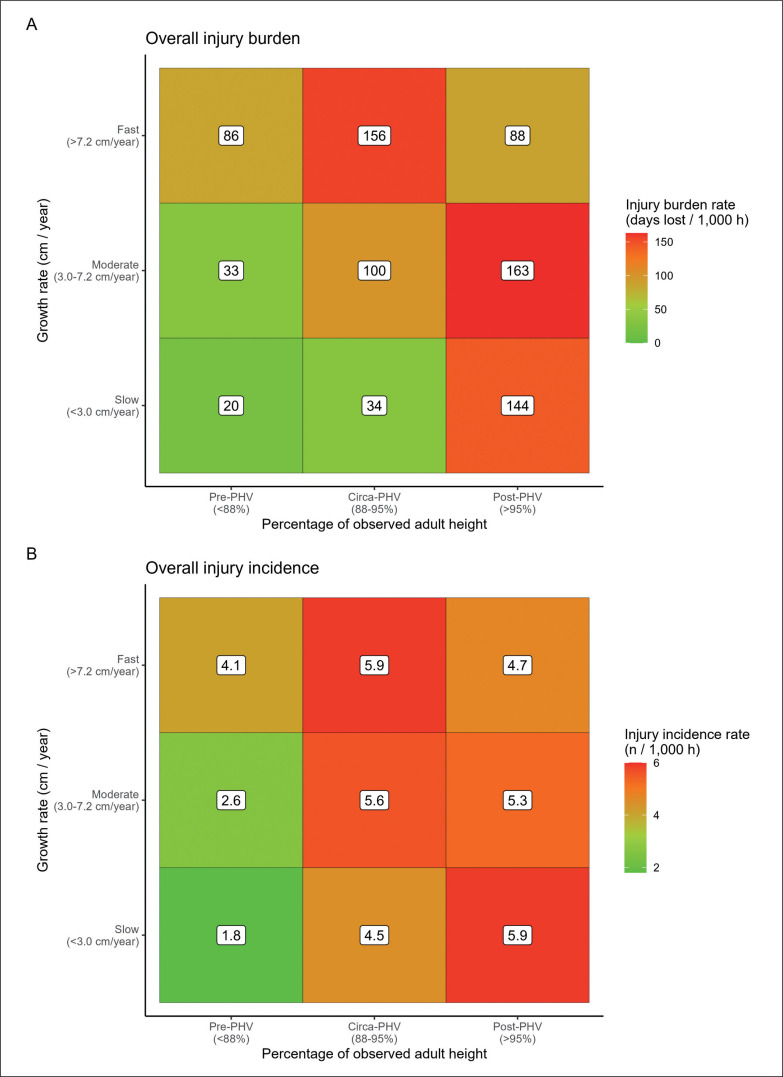
Overall injury burden (A) and incidence (B) according to growth rate and percentage of observed adult height.

Overall injury incidence was 1.65- and 2.38-times greater in pre-PHV players with fast growth rates (4.1 injuries/1000 h, 95% CI: 2.8–5.2/1000 h) compared to players growing moderately (2.6 injuries/1000 h, 95% CI: 1.9–3.0/1000 h) and slowly (1.8 injuries/1000 h, 95% CI: 0.8–3.1/1000 h), respectively. Similarly, overall injury burden in pre-PHV players growing fast (86 days lost/1000 h, 95% CI: 59–125/1000 h) was 2.9- and 4.4-times higher compared to pre-PHV players with moderate (33 days lost/1000 h, 95% CI: 25–44/1000 h) and slow (20 days lost /1000 h, 95% CI: 8–46/1000 h) growth rates (Figure 1).

**FIG. 2:**
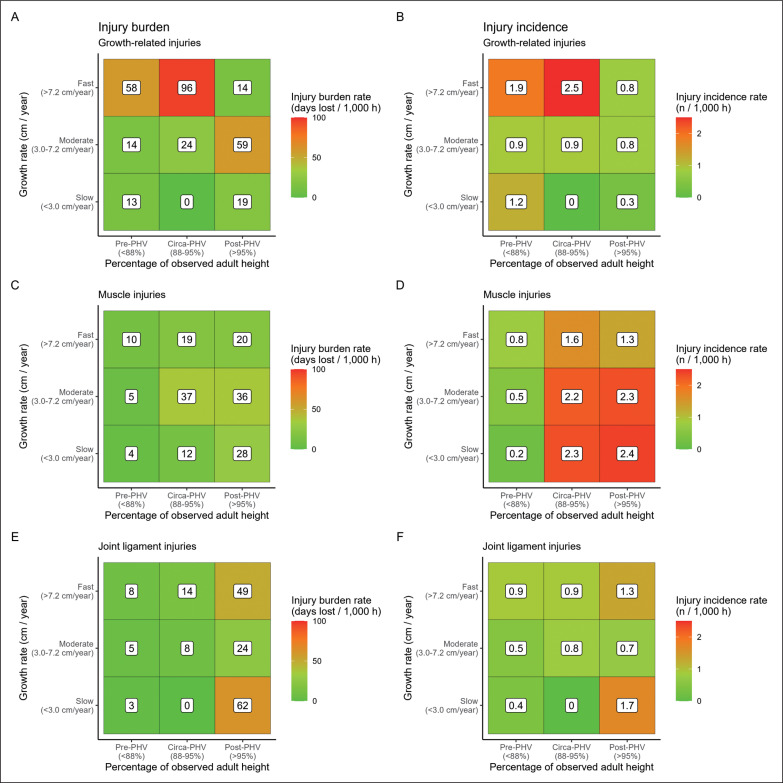
Injury burden and incidence of growth-related (A, B), muscle (C, D) and joint/ligament injuries (E, F) according to growth rate and percentage of observed adult height.

**FIG. 3:**
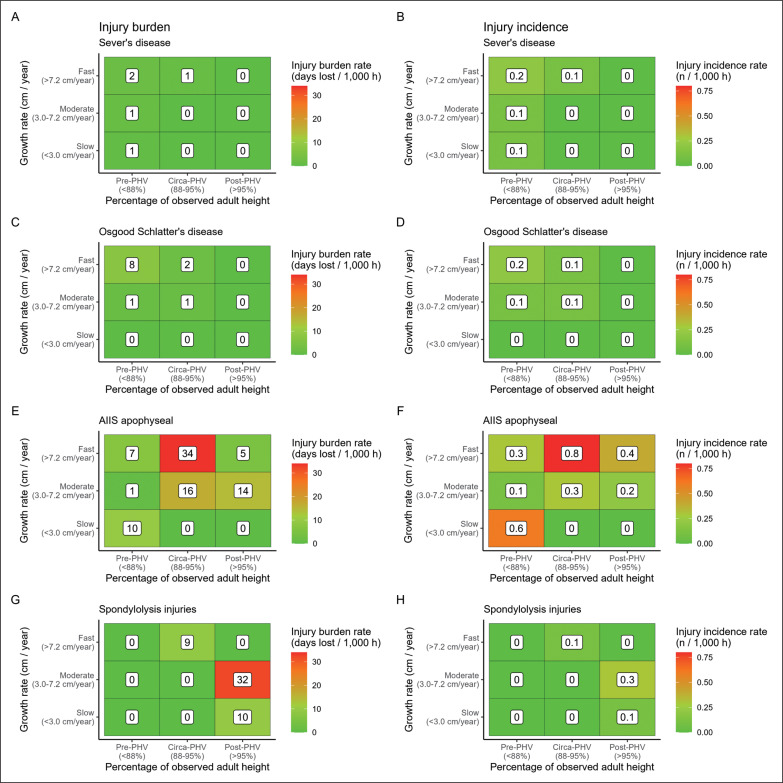
Injury burden and incidence of specific growth-related injuries according to growth rate and percentage of observed adult height.

Concerning growth-related injuries, in the pre-PHV period, incidence and burden were 2.5- and 5.4-times higher in players with fast growth rates (1.9 injuries/1000 h, 95% CI: 1.2–2.8/1000 h and 58 days lost/1000 h, 95% CI: 34–101/1000 h) compared to players growing moderately (0.9 injuries/1000 h, 95% CI: 0.5–1.1/1000 h and 14 days lost/1000 h, 95% CI: 9–23/1000 h). In the same line, circa-PHV players growing fast showed 2.8- and 3.4-times greater injury incidence and burden (2.5 injuries/1000 h, 95% CI: 1.7–3.2/1000 h and 96 days lost/1000 h, 95% CI: 68–136/1000 h) compared to players growing moderately (0.9 injuries/1000 h, 95% CI: 0.4–1.6/1000 h and 24 days lost/1000 h, 95% CI: 10–57/1000 h) (Figure 2). In post-PHV, growth-related injury incidence was 2.4-times higher in players growing fast (0.8 injuries/1000 h, 95% CI: 0.2–3.4/1000 h) compared to players growing slowly (0.3 injuries/1000 h, 95% CI: 0.2–0.5/1000 h) (Figure 2). Concerning injury risk for specific growth-related injuries, pre-PHV players growing fast showed a 4.4-times higher Osgood-Schlatter´s disease incidence (0.2 injuries/1000 h, 95% CI: 0.1–1.2/1000 h) compared to players growing moderately (0.1 injuries/1000 h, 95% CI: 0.1–0.3/1000 h) (Figure 3). Moreover, post-PHV players growing fast had a higher incidence of anterior inferior iliac apophyseal injuries (0.4 injuries/1000 h, 95% CI: 0.1–3.9/1000 h) compared to players growing slowly (0.2 injuries/1000 h, 95% CI: 0.1–0.2/1000 h) (RR: 257.9) (Figure 3).

Significant differences for incidence and burden of muscle injuries were not found between any of the growth rates groups in pre-, circa- and post-PHV periods. Nevertheless, the incidence and burden of joint/ligament injuries were 2.4 and 2.6-times greater in post-PHV players growing slowly (1.7 injuries/1000 h, 95% CI: 1.3–2.1/1000 h and 62 days lost/1000 h, 95% CI: 47–81/1000 h) compared to those growing moderately (0.7 injuries/1000 h, 95% CI: 0.4–1.1/1000 h and 24 days lost/1000 h, 95% CI: 12–45/1000 h) (Figure 2).

## DISCUSSION

This is the first research studying the main and interactive effects of growth rate and maturity status on risk for specific types of injuries in academy football. We improved upon the limitations of previous research by using longitudinal height data from childhood to adulthood to estimate daily growth rate and percentage of observed adult height to study how growth rate influences overall and specific incidence and burden in pre-, circa- and post-PHV periods. All in all, our results demonstrated that players with higher growth-rates were at higher risk for growth-related injuries independently to the somatic maturation status. Besides, slow growth rate post-PHV players had a higher incidence and burden of joint/ligament injuries.

The major finding of this study is that growth-rate affects overall injury risk in pre-PHV period, which highlights the importance of regular growth monitoring from an early age. Multiple injury mechanisms may explain increased injury risk in pre-PHV players with fast growth rates. Rapid growth might lead to larger changes to limb length, limb mass, and moments of inertia [24], alterations in motor control [5], which may adversely impact injury risk. Rapid longitudinal skeletal growth is also associated with a temporary decrease in bone mineral density and weakness of the epiphyseal growth plates [25], and may facilitate the appearance growth-related conditions [26]. Considering that the growth-related injuries have the highest incidence [15] and burden [17] in pre-PHV period, increased growth-related injury risk in players growing fast might have contributed to increased overall injury risk. Another reason that could explain the higher risk in pre-PHV players growing fast, might be that pre-PHV players with faster growth-rates could be earlier maturers [4]. Players maturing earlier usually have faster growth rates [4] and might be physically superior to their peers [27]. Thus, they may develop a more physical way of playing football [28] exposing them to a higher injury risk in pre-PHV [17]. Future research should consider maturity timing when studying the interaction of growth-rate, maturity, and injury risk.

The results of the current investigation showed a higher incidence and burden of growth-related injuries in players with fast growth rates compared to those growing moderately in pre- and circa-PHV, and a higher incidence in players with moderate growth rates compared to those growing slowly in post-PHV. The small number of playing growing slowly (< 3.5 cm/year) in pre- and circa-PHV might have led to not finding significant differences in those groups. In the same line, the lack of players growing fast in post-PHV period may explain why significant differences compared to this group were not found; however, players growing quick had the highest incidence of growth-related injuries in this period. The combination of altered sensorimotor mechanisms and motor control [5] and vulnerability of apophyses [25] might result in increased injury growth-related injury incidence and burden in players growing fast [26], which is in line with previous research by Wik et al. [12]. Besides, it was not surprising to find that faster growth rates lead to higher risk for growth-related injuries in all pre-, circa- and post-PHV periods, as previous research has already shown that these injuries can occur all along the maturation process [15, 17]. Interestingly, our results showed that growth-rate affected risk for specific types of growth-related injuries differently according to maturity status, which is in accordance with the distal to proximal pattern of growth-related injuries found in previous research [15, 17].

No significant results between incidence and burden of muscle injuries were found between growth rate groups (fast *vs*. moderate *vs*. slow) in pre-, circa- and post-PHV. These results are in line with previous research by Wik et al. [12], who only found an association between growth and risk of bone and growth-plate injuries. More research is needed to better understand if neuromuscular alterations that appear around PHV [29] are related to the higher muscle and joint/ligament injury risk in circa- and post-PHV periods [15, 17].

Concerning injury risk for joint/ligament injuries, players growing slowly had a higher incidence and burden compared to those with fast/moderate growth rates in post-PHV. Our results are in accordance with recent results found by Monasterio et al. [17], who found a higher injury burden for joint/ligament injuries in adult players (growth rate < 1 cm/year), compared to post-PHV players who may have been growing at higher rates. Considering that post-PHV players growing slow may be more mature (and older) than players growing fast and moderately, our results might be explained by the accumulation of multiple seasons of training and competition throughout their careers [30], with previous injury increasing the risk of subsequent injury [31].

### Practical application

In light of the results above, we recommend academy practitioners to measure players height every 3–4 months [32] to model individual growth curves and estimate growth velocities. In order to monitor maturity status (percentage of predicted adult height), an x-ray of the hand-wrist complex is considered the best method to use [4]. However, exposure to low-level radiation, the need for specialised equipment and trained technicians makes it impractical in academies. Thus, other non-invasive and cost-efficient alternatives such as the Khamis-Roche method (somatic maturity) [33] or SonicBone BAUSPORT system (skeletal maturity via ultrasound) [34] could be used to estimate percentage of adult height.

**FIG. 4:**
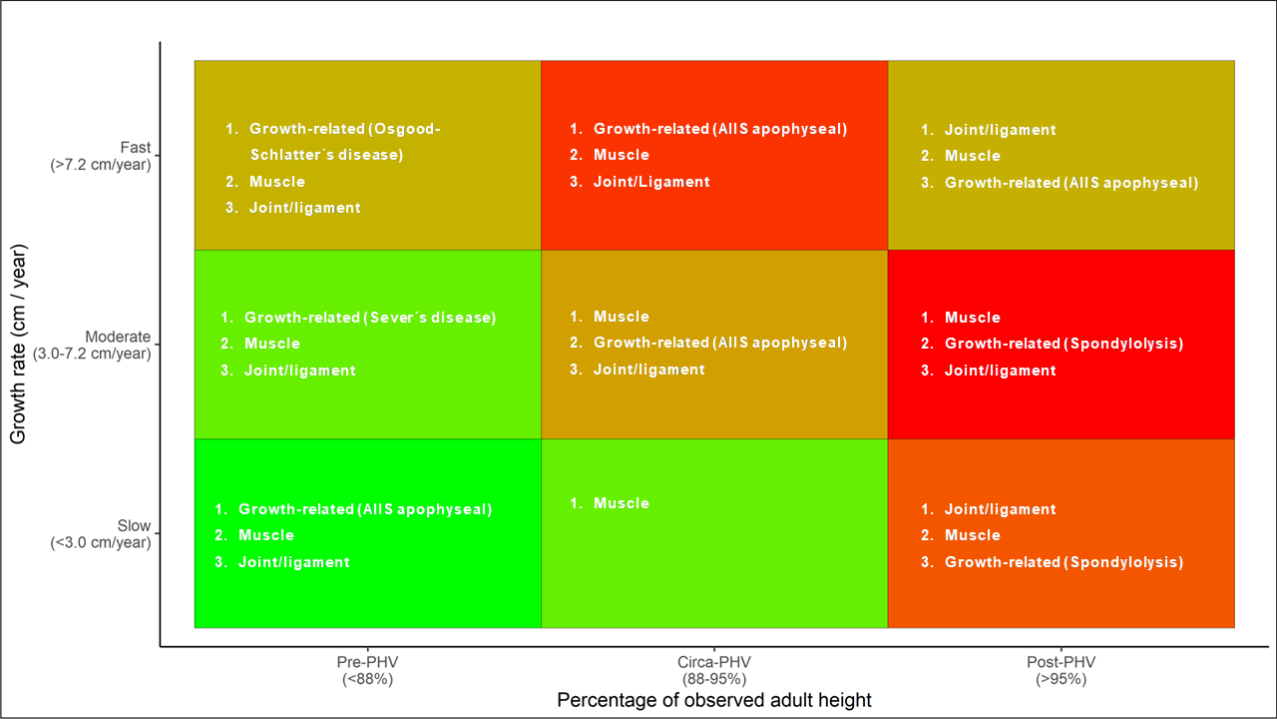
Ranking for most burdensome type of injuries according to growth rate and maturity status.

Once estimated each player´s growth rate and maturity status (pre-, circa-, post-PHV), Figure 4 could be used in a practical setting to identify players at higher risk (red colour). This figure will be helpful to facilitate the interpretation of our results to key decision-makers in football academies (players, coaches, and directors), who may be unfamiliar with scientific figures and data analysis. As a result, it may improve communication with key decision-makers and increase their engagement in injury management strategies. Practitioners may choose the adjust training content and training and competition load during periods of heightened injury risk (i.e., adolescent growth spurt) to mitigate injury risk. Jan Willem Teunnisen, a former movement scientist at Ajax Football Club describes an innovative bio-banding (i.e., maturity matching) strategy whereby the player’s entering the adolescent growth spurt were prescribed a training programme that emphasised core strength, balance, coordination, the re-training of fundamental and sport-specific motor skills, and the maintenance mobility, in addition to a reduction in training and competition load [35]. The purpose of this programme was to reduce injury risk and aid transition through this phase of development.

The growth/maturity heat maps also highlight the most burden-some injuries [36] in each quadrant and may guide practitioners to design targeted injury risk reduction strategies. As shown in previous research [15, 17], reducing the impact of growth-related injuries seems vital in pre- and circa-PHV periods. Further, this research high-lights the need for special attention to those players growing at velocities > 7.2 cm/year. Strategies such as controlling week-to-week changes in load [11, 37], changing training content [35] or monitoring symptoms of musculoskeletal complaints to detect early growth-related conditions [38] may be of the utmost importance in those players. Due to the distal to proximal patterns of growth-related injuries, special awareness to symptoms in the ankle/knee should be taken in pre-PHV period, while focussing on complaints on the hip/pelvis and lower back is essential in circa- and post-PHV, respectively. On the other hand, reducing the impact of spondylolysis, muscle and joint/ligament injuries seems vital in post-PHV. For instance, controlling training load [37] or neuromuscular training programmes [39] might be beneficial to reduce injury risk during this period.

### Methodological considerations

The principal strength of this study is its longitudinal design over two decades, which allowed to model growth rates and estimate daily growth rate and percentage of observed adult height. This research has improved on previous data that recorded growth during short periods [8–12], not allowing to account for the non-linear characteristic of growth [16]. Besides, this study used percentage of observed adult height as a maturity status indicator, while previous studies calculated percentage of predicted adult height [11, 14]. Most importantly, this is the first study investigating the interaction between growth rate and injury risk (incidence and burden) for specific types of injuries according to maturity status (pre-, circa- and post-PHV).

However, the limitations of the current investigation should also be noted. Firstly, we did not account for individual exposure. Thus, as suggested by the latest international Olympic Committee consensus statement [21], exposure was estimated based on the number and duration of matches and training sessions, squad size and the number of players on the pitch in each category. Besides, our findings apply to a single elite soccer academy, and only players who attained adult height were included in the study. Considering that injuries have a negative impact on academy progression [2], players who sustained severe injuries may have been missed. Moreover, injury data was analysed retrospectively and classification by the FIFA Consensus was not considered since the start of the study. Further, there were no protocols to check intra- and inter-tester reliability of all the doctors that recorded injuries during the whole study period.

Further, many factors such as equipment used to measure height or diagnose players’ injuries, preventive strategies and training content might have changed over the study period and were not controlled for in analyses. Another limitation is that our sample size was not large enough to detect association with all specific injuries [40], and the limited number of specific injuries resulted in wide confidence intervals for the injury incidence and burden of many injuries. Thus, we only studied the most frequent injuries in our dataset. Future studies should build on this work by conducting multi-team collaborative studies with a sufficiently powered sample size.

## CONCLUSIONS

Our results demonstrated that players with higher growth-rates were at higher risk for growth-related injuries in all pre-, circa- and post-PHV periods. A higher incidence and burden for joint/ligament injuries in players with slow growth rate post-PHV compared to players with moderate growth rate was found. Thus, practitioners in football academies should consider the combined effects of growth rate and maturity status when designing targeted injury risk reduction strategies.
